# *BRCA1/2* Mutations and Cardiovascular Function in Breast Cancer Survivors

**DOI:** 10.3389/fcvm.2022.833171

**Published:** 2022-02-15

**Authors:** Biniyam G. Demissei, WenJian Lv, Nicholas S. Wilcox, Karyn Sheline, Amanda M. Smith, Kathleen M. Sturgeon, Chris McDermott-Roe, Kiran Musunuru, Bénédicte Lefebvre, Susan M. Domchek, Payal Shah, Bonnie Ky

**Affiliations:** ^1^Division of Cardiology, Department of Medicine, Perelman School of Medicine at the University of Pennsylvania, Philadelphia, PA, United States; ^2^Department of Public Health Sciences, Pennsylvania State College of Medicine, Hershey, PA, United States; ^3^Abramson Cancer Center, Perelman School of Medicine at the University of Pennsylvania, Philadelphia, PA, United States; ^4^Division of Hematology and Oncology, Department of Medicine, Perelman School of Medicine at the University of Pennsylvania, Philadelphia, PA, United States; ^5^Department of Biostatistics, Epidemiology and Informatics, Perelman School of Medicine at the University of Pennsylvania, Philadelphia, PA, United States

**Keywords:** anthracycline, *BRCA1/2*, breast cancer, cardiomyocyte, heart failure, HER2 therapy

## Abstract

**Objective:**

Animal models suggest that *BRCA1/2* mutations increase doxorubicin-induced cardiotoxicity risk but data in humans are limited. We aimed to determine whether germline *BRCA1/2* mutations are associated with cardiac dysfunction in breast cancer survivors.

**Methods:**

In a single-center cross-sectional study, stage I-III breast cancer survivors were enrolled according to three groups: (1) *BRCA1/2* mutation carriers treated with doxorubicin; (2) *BRCA1/2* mutation non-carriers treated with doxorubicin; and (3) *BRCA1/2* mutation carriers treated with non-doxorubicin cancer therapy. In age-adjusted analysis, core-lab quantitated measures of echocardiography-derived cardiac function and cardiopulmonary exercise testing (CPET) were compared across the groups. A complementary *in vitro* study was performed to assess the impact of *BRCA1* loss of function on human induced pluripotent stem cell-derived cardiomyocytes (iPSC-CMs) survival following doxorubicin exposure.

**Results:**

Sixty-seven women with mean (standard deviation) age of 50 (11) years were included. Age-adjusted left ventricular ejection fraction (LVEF) was lower in participants receiving doxorubicin regardless of *BRCA1/2* mutation status (*p* = 0.03). In doxorubicin-treated *BRCA1/2* mutation carriers and non-carriers, LVEF was lower by 5.4% (95% CI; −9.3, −1.5) and 4.8% (95% CI; −9.1, −0.5), respectively compared to carriers without doxorubicin exposure. No significant differences in VO_2max_ were observed across the three groups (p_overall_ = 0.07). Doxorubicin caused a dose-dependent reduction in viability of iPSC-CMs *in vitro* without differences between *BRCA1* mutant and wild type controls (*p* > 0.05).

**Conclusions:**

*BRCA1/2* mutation status was not associated with differences in measures of cardiovascular function or fitness. Our findings do not support a role for increased cardiotoxicity risk with *BRCA1/2* mutations in women with breast cancer.

## Introduction

*BRCA1/2* genes play a critical role in multiple cellular processes governing genome stability including DNA repair. In addition to suppressing tumor growth, *BRCA1/2* genes may play a role in the maintenance of cardiomyocyte survival and function (1). In animal models, loss of cardiomyocyte-specific *BRCA1/2* is associated with DNA damage, apoptosis, cardiac dysfunction, and cardiac mortality following doxorubicin exposure (1, 2). *BRCA1/2* genes may potentially mitigate against anthracycline-induced genotoxic stress and cardiomyocyte apoptosis and thus serve a cardioprotective role. However, whether these preclinical findings translate to humans is unclear (3–5).

In a single-center, cross-sectional study, we investigated differences in cardiac function and cardiopulmonary fitness through comprehensive phenotyping of breast cancer survivors with and without *BRCA1/2* mutations. Furthermore, we performed an *in vitro* study using human induced pluripotent stem cell-derived cardiomyocytes (iPSC-CMs) to assess the impact of *BRCA1* loss on cardiomyocyte survival following doxorubicin exposure.

## Methods

### Study Population

The Genetics and Heart Health After Cancer Therapy (Gene-HEART) study (NCT03510689) evaluated stage I-III breast cancer survivors older than 18 years old treated at the University of Pennsylvania Abramson Cancer Center (Philadelphia, Pennsylvania). Three groups of breast cancer survivors were enrolled at least ~12 months after initiation of chemotherapy. These included: (1) *BRCA1/2* mutation carriers treated with 240 mg/m^2^ of doxorubicin; (2) *BRCA1/2* mutation non-carriers treated with 240 mg/m^2^ of doxorubicin; and (3) *BRCA1/2* mutation carriers treated with non-doxorubicin cancer therapy. Exclusion criteria included stage IV disease, genetic testing confirming a variant of unknown significance or benign polymorphism in *BRCA1/2* genes, contraindications to VO_2_ testing, or pregnancy. The study was approved by the University of Pennsylvania Institutional Review Board, and all participants provided written informed consent.

### Echocardiography Quantitation

Participants underwent comprehensive phenotyping with echocardiography-derived measures of systolic and diastolic cardiac function (TomTec Imaging Systems platform, Unterschleissheim, Germany). Quantitative echocardiography was performed by a single blinded observer at the University of Pennsylvania Center for Quantitative Echocardiography (Philadelphia, PA). Intra-observer coefficients of variation were 4.5, 9.0, and 9.7% for LVEF, longitudinal strain, and circumferential strain, respectively, and 4–5% for mitral inflow and tissue Doppler velocities. The absolute values of longitudinal and circumferential strain are presented, whereby a greater absolute value represents improved function.

### Cardiopulmonary Exercise Testing

Cardiopulmonary exercise testing (CPET) was performed based on the modified Bruce protocol with continuous measurement of breath-by-breath gas sampling oxygen consumption (VO_2_) using a calibrated metabolic cart (ParvoMedics TrueOne® 2400, Sandy, UT).

### Statistical Analysis

Baseline characteristics were summarized according to exposure group using proportions for categorical variables while mean (standard deviation [SD]) and median (quartile 1 [Q1], quartile 3 [Q3]) were utilized for normally and non-normally distributed continuous variables, respectively. In cross-sectional analysis, measures of cardiac function and cardiopulmonary fitness were compared across the three groups. Age-adjusted marginal means and their respective 95% confidence intervals (CI) were estimated for each parameter, and group differences were tested using analysis of covariance. We performed sensitivity analysis by excluding HER2-positive breast cancer participants who received trastuzumab to determine the potential effect of targeted cardiotoxic cancer therapy. Statistical significance was evaluated at a two-sided alpha level of 5%. Analyses were performed using R 3.4.0 (R Foundation for Statistical Computing, Vienna, Austria).

### Experimental Design

For the *in vitro* study, a premature stop codon was introduced via CRISPR-Cas9 into one *BRCA1* allele in a healthy donor-derived iPSC line (Supplementary Figure 1). Cells which were transfected but not mutated were retained as wild type controls. *BRCA1* mutant and wild type iPSCs were differentiated into cardiomyocytes (iPSC-CMs) using an established protocol (6). At 25 days post-differentiation, cardiomyocytes received varying concentrations of doxorubicin (1–500 nM). Cell viability was assessed using alamarBlue Cell Viability Reagent.

## Results

The mean (SD) age of the 67 breast cancer survivors in the study cohort was 50 (11) years, 87% were White and 64% had stage II/III disease. The median (Q1, Q3) time from diagnosis at enrollment was 6 (3, 7) years. Table 1 summarizes baseline characteristics according to *BRCA1/2* status and doxorubicin exposure.

**Table 1:** Baseline characteristics according to exposure group.

**Baseline characteristics**	***BRCA1/2*** **Carriers,** **doxorubicin** **(***n*** = 39)**	***BRCA1/2*** **Carriers,** **no doxorubicin** **(***n*** = 14)**	***BRCA1/2*** **Non-carriers,** **doxorubicin** **(***n*** = 14)**
Age at study enrollment (years)	46.0 (10.1)	57.0 (10.1)	54.6 (7.9)
**Race**			
White	30 (76.9)	14 (100)	14 (100)
Black	4 (10.3)	0 (0)	0 (0)
Asian	2 (5.1)	0 (0)	0 (0)
Unknown	3 (7.7)	0 (0)	0 (0)
Years from breast cancer diagnosis	5 (3, 8)	7 (6, 7)	4 (3, 6)
**Breast cancer stage**			
I	11 (28.9)	10 (71.4)	3 (21.4)
II/III	27 (71.1)	4 (28.6)	11 (78.5)
**Disease site**			
Left	18 (46.1)	6 (42.9)	8 (61.5)
Right	20 (51.3)	8 (57.1)	5 (38.5)
Lymph nodes only	1 (2.6)	0 (0)	0 (0)
**HER2 status**			
Positive	2 (5.1)	1 (7.7)	3 (21.4)
**ER status**			
Positive	19 (48.7)	11 (84.6)	9 (64.3)
**PR status**			
Positive	20 (51.3)	11 (84.6)	8 (57.1)
Triple negative breast cancer	18 (46.2)	1 (7.7)	4 (28.6)
Trastuzumab with or without pertuzumab	3 (7.7)	0 (0)	3 (21.4)
Tamoxifen	11 (29.3)	7 (53.8)	3 (21.4)
Aromatase inhibitors	18 (47.4)	9 (69.2)	6 (42.9)
Radiation therapy	18 (51.4)	5 (35.7)	9 (69.2)
Mastectomy	29 (78.4)	11 (84.6)	7 (50.0)
Bilateral salpingo-oophorectomy	28 (75.5)	12 (85.7)	2 (15.4)
Body mass index (Kg/m^2^)	27.4 (5.7)	26.4 (5.1)	24.4 (2.8)
Systolic blood pressure (mmHg)	118.6 (3.6)	117.7 (17.8)	116.5 (11.1)
Current or past smoking	12 (30.8)	3 (23.1)	6 (42.8)
Diabetes mellitus	1 (2.6)	0 (0)	1 (7.1)
Hypertension	8 (20.5)	2 (14.3)	2 (14.3)
Hyperlipidemia	10 (25.6)	4 (28.6)	3 (21.4)
ACEI/ARBs or Beta-blockers	4 (10.3)	2 (16.7)	2 (14.3)
Statins	6 (15.4)	2 (16.7)	1 (7.1)

*ACE, Angiotensin converting enzyme; ARB, Angiotensin receptor blocker*.

*For baseline characteristics, categorical variables are summarized using count (proportion); age, body mass index and systolic blood pressure are summarized using mean (standard deviation); Years from diagnosis is summarized using median (Q1, Q3)*.

**Figure 1:**
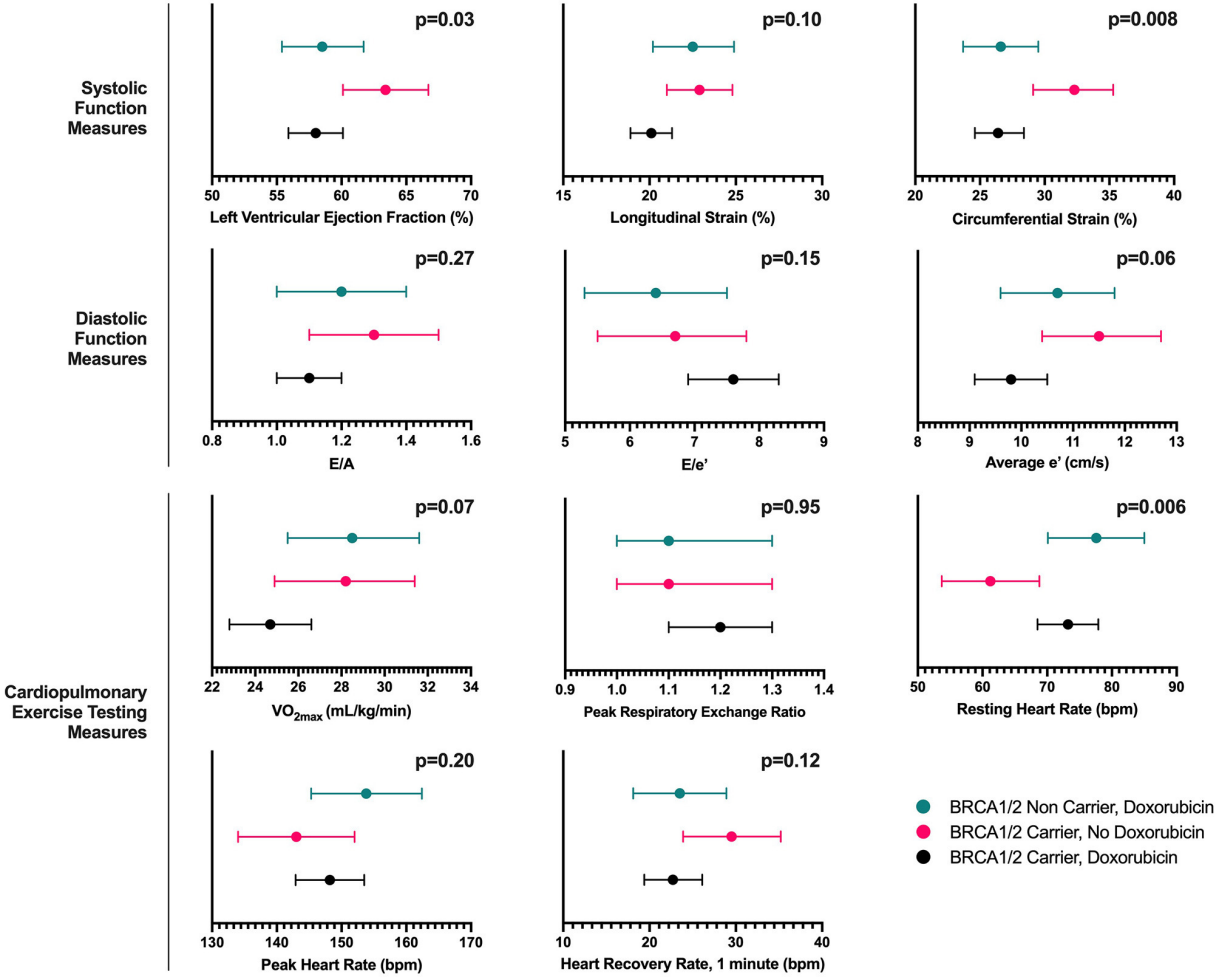
Age-adjusted marginal mean (95% Confidence Interval) estimates of echocardiography and cardiopulmonary exercise testing measures according to exposure group. The figure presents the age-adjusted marginal mean (95% confidence interval) estimates based on analysis of covariance for measures of systolic function, diastolic function and cardiopulmonary exercise testing according to exposure group including (a) *BRCA1/2* mutation carriers exposed to doxorubicin, (b) *BRCA1/2* mutation carriers not exposed to doxorubicin, and (c) *BRCA1/2* mutation non-carriers exposed to doxorubicin. For longitudinal and circumferential strain, absolute values are presented, where by a higher value represents greater function.

Participants were assessed at a median (Q1, Q3) of 4 (2, 6) years after completion of chemotherapy. The age-adjusted left ventricular ejection fraction (LVEF) was significantly lower in participants treated with doxorubicin, regardless of *BRCA1/2* mutation status (*p* = 0.03). In doxorubicin-treated *BRCA1/2* mutation carriers and non-carriers, estimated differences were lower by 5.4% (95% CI; −9.3, −1.5) and 4.8% (95% CI; −9.1, −0.5), respectively, compared to carriers without doxorubicin exposure. These findings were consistent across additional cardiac function measures including circumferential and longitudinal strain, although less pronounced for the latter. There were no differences in diastolic function measures E/A, e', and E/e' (Figure 1, Supplementary Table 1). These findings remained consistent in a sensitivity analysis excluding 6 participants who had received HER2-targeted therapy (Supplementary Table 2).

Among CPET measures, the age-adjusted resting heart rate was significantly higher in the doxorubicin-treated groups regardless of *BRCA1/2* status. However, we did not find significant differences across the three groups in VO_2max_, peak heart rate or peak respiratory exchange ratio (Figure 1, Supplementary Table 1). Similar findings were observed in a sensitivity analysis excluding participants who received HER2-targeted therapy (Supplementary Table 2). We also performed additional sensitivity analysis comparing echocardiography and CPET measures across the groups using a non-parametric test (i.e., Kruskal-Wallis test) and the findings were largely similar.

**Figure 2:**
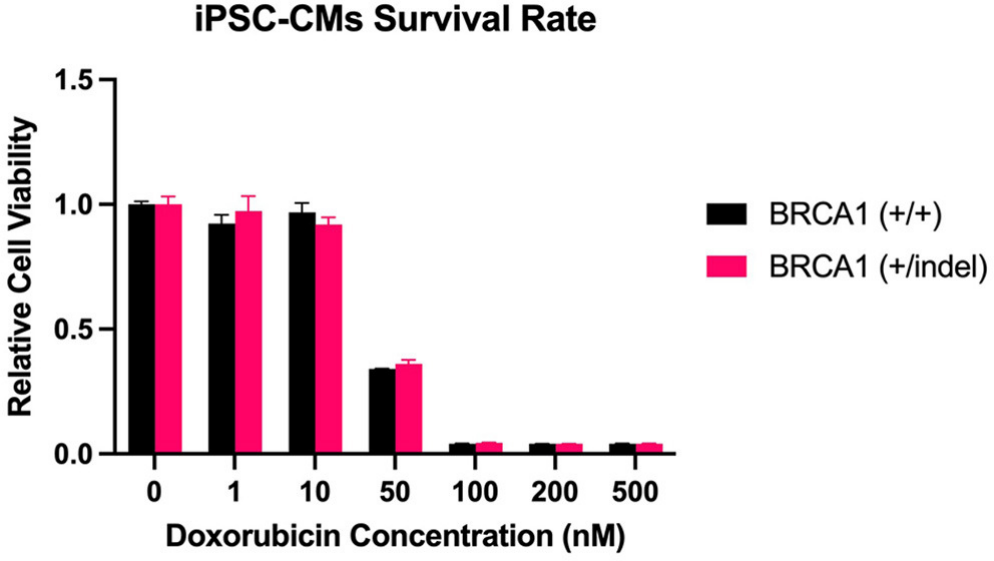
*BRCA1* mutation and cardiomyocyte cell viability following doxorubicin. The figure presents comparisons of cell viability between *BRCA1* mutant [*BRCA1* (+/indel)] and wild type [*BRCA1* (+/+)] human induced pluripotent stem cell-derived cardiomyocytes (iPSC-CMs) following exposure to 1–500 nM doxorubicin concentration.

*In vitro*, doxorubicin caused a dose-dependent reduction in cell viability with no differences between *BRCA1* mutant and wild type iPSC-CMs (p>0.05). Estimates of cell viability (doxorubicin concentration) in *BRCA1* mutant compared with wild type iPSC-CMs were 97.3 vs. 92.4% (1 nM), 91.9 vs. 96.7% (10 nM), 36.0 vs. 34.0% (50 nM), 4.4 vs. 4.1% (100 nM), and 4.1 vs. 4.1% (500 nM) (Figure 2).

## Discussion

Overall, our results suggest that women with breast cancer who have *BRCA1/2* mutations are not at increased risk of anthracycline-induced cardiotoxicity relative to those with sporadic breast cancer. This is based on several lines of evidence. First, although we observed significantly lower left ventricular systolic function in breast cancer survivors treated with doxorubicin compared to those without doxorubicin exposure, we did not find differences in age-adjusted estimates of echocardiography-derived measures of systolic or diastolic dysfunction according to germline *BRCA1/2* mutation status. Second, there were no significant differences in cardiopulmonary fitness measures as determined by CPET based on *BRCA1/*2 status. Third, complementary *in vitro* experiments showed a comparable dose-dependent reduction in cell viability in both loss of function *BRCA1* mutant and wild type iPSC-CMs receiving doxorubicin.

*BRCA1/2* mutations may be associated with increased risk of doxorubicin cardiotoxicity, but human data are limited. One prior exploratory study of 401 patients, including *232 BRCA1* and 159 *BRCA2* mutation carriers, showed an increased risk of heart failure based on self-reported symptoms elicited on an anonymous survey, relative to historical controls drawn from the general population (3). In this study, however, the authors were unable to verify reported symptoms using objective confirmatory data such as echocardiogram reports in most participants, and there was no direct comparator control group. In contrast, two other studies found no significant differences between rates of cardiomyopathy in *BRCA1/2* mutation carriers vs. wild type controls receiving anthracyclines, though each had limitations (4, 5). One prospective study was underpowered to assess for differences in cardiac dysfunction between groups, excluded participants with hypertension or those who received trastuzumab, and did not demonstrate expected LVEF declines among *BRCA1/2* mutation carriers receiving anthracyclines (4). A second retrospective study only evaluated the incidence of either asymptomatic decline in LVEF to <50% or heart failure and lacked detailed assessment of subclinical measures of cardiovascular function (5). Only a minority of participants included in the study underwent follow-up LVEF assessment after completion of anthracycline therapy limiting the ability to detect asymptomatic declines in cardiac function. Our study fills an important evidence gap by comprehensively characterizing cardiac function using both quantitative echocardiography and CPET and performing complementary *in vitro* experiments using iPSC-CMs.

Our human data contrast with the results of murine studies, where loss of *BRCA1/2* in cardiomyocytes was associated with worse cardiac function and increased mortality following doxorubicin exposure (1, 2). There are several possible explanations for this. First, significant differences exist in the physiology of human and murine cardiomyocytes including calcium cycling, expression of ion channels, energetics, and myofilament composition (7). Second, cardiomyocyte specific *BRCA1/2* knockouts in mice are biologically distinct from inherited germline *BRCA1/2* mutations in humans. Third, mice used in preclinical studies were either exclusively male or the sex was not disclosed and administered relatively higher anthracycline doses compared to standard chemotherapy dosing regimens, potentially contributing to discrepancies in results (1, 2).

Our study has limitations. Though the study is one of the few studies to date to assess the impact of *BRCA1/2* mutations on detailed measures of cardiac function in breast cancer patients receiving anthracyclines, statistical power was limited due to sample size. Our analyses were adjusted for age alone given the relatively small sample size, and confounding remains possible. Furthermore, limitations related to unequal group sizes should be considered. Our *in vitro* experiments do not incorporate hemodynamic or neurohormonal stressors inherent to *in vivo* studies, which may diminish observed differences, particularly with respect to *BRCA1* status (8). In addition, we focused on cell viability in the *in vitro* study, and other measures related to iPSC-CM structure and function were not evaluated.

In conclusion, we present both detailed phenotypic characterization of cardiac function, including echocardiography and CPET, in breast cancer survivors with and without *BRCA1/2* mutations treated with anthracyclines, and *in vitro* characterization using anthracycline-treated, wild type vs. gene-modified human iPSC-CMs with a loss of function mutation in *BRCA1*. Overall, we found no strong evidence to support associations between *BRCA1/2* mutations and anthracycline-induced cardiac dysfunction based on echocardiography, CPET or *in vitro* data. Our study fills an important evidence gap and adds support to the lack of increased cardiotoxicity risk in breast cancer patients with *BRCA1/2* mutations.

## Data Availability Statement

The original contributions presented in the study are included in the article/Supplementary Materials, further inquiries can be directed to the corresponding author.

## Ethics Statement

The studies involving human participants were reviewed and approved by the University of Pennsylvania Institutional Review Board. The patients/participants provided their written informed consent to participate in this study.

## Author Contributions

BK, PS, and AS contributed to conception and design of the study. BK, AS, KS, and KMS contributed to clinical data collection. WL, CM-R, BK, and KM contributed to the design and execution of the *in vitro* study. BD and BK performed statistical analysis. BD, NW, and BK wrote the first draft of the manuscript. All authors contributed to manuscript revision, read, and approved the submitted version.

## Funding

Research reported in this publication was supported by the National Center for Advancing Translational Sciences of the NIH under Award Number UL1TR001878 and R01HL118018 (BK) and R35HL145203 (KM). KMS was supported by grant from the National Center for Advancing Translational Sciences (5UL1TR002014 and 5KL2TR002015).

## Author Disclaimer

The content is solely the responsibility of the authors and does not necessarily represent the official views of the NIH.

## Conflict of Interest

The authors declare that the research was conducted in the absence of any commercial or financial relationships that could be construed as a potential conflict of interest.

## Publisher's Note

All claims expressed in this article are solely those of the authors and do not necessarily represent those of their affiliated organizations, or those of the publisher, the editors and the reviewers. Any product that may be evaluated in this article, or claim that may be made by its manufacturer, is not guaranteed or endorsed by the publisher.
