# Supplementary material for: BRCA1/2 Mutations and Cardiovascular Function in Breast Cancer Survivors
Source: Front Cardiovasc Med. 2022 Feb 15;9:833171. doi: 10.3389/fcvm.2022.833171 (PMC8885808; doi:10.3389/fcvm.2022.833171)
Supplement: Supplementary file 1 [file Data_Sheet_1.docx]

**Supplementary Material**


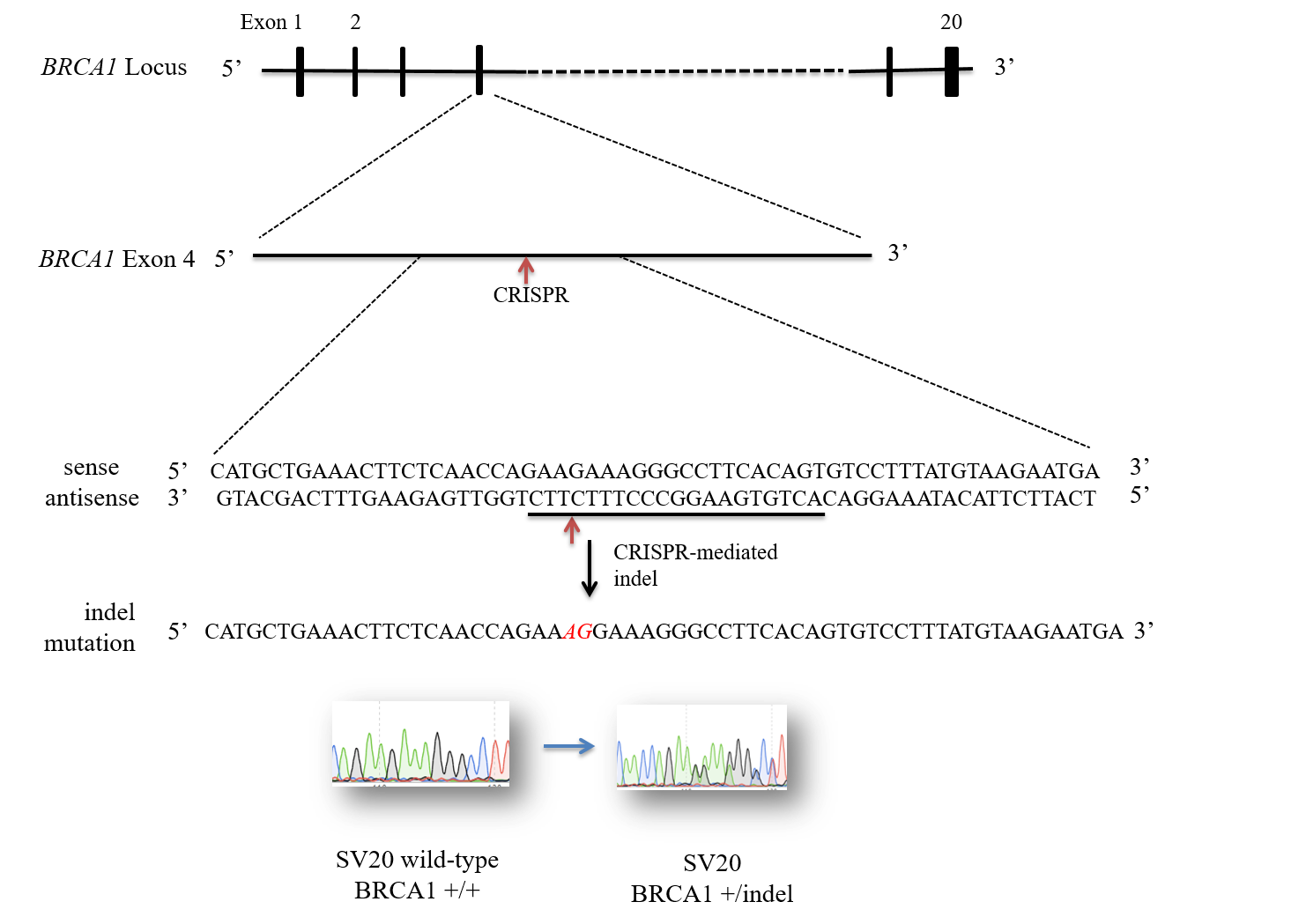


**Supplementary Figure 1**: CRISPR-Cas9-based introduction of a premature stop codon into a *BRCA1* allele from a healthy donor-derived human induced pluripotent stem cell-derived cardiomyocyte

**Supplementary Table 1**: Age-adjusted marginal mean (95% CI) estimates of echocardiography and cardiopulmonary exercise testing measures according to exposure group

| **Variable** | ***BRCA1/2* Carriers, Doxorubicin** | ***BRCA1/2* Carriers, No Doxorubicin** | ***BRCA1/2* Non-carriers, Doxorubicin** | **P-value** |
| --- | --- | --- | --- | --- |
| ***Systolic function*** |  |  |  |  |
| Left ventricular ejection fraction (%) | 58.0 (55.9,60.1) | 63.4 (60.1,66.7) | 58.5  (55.4,61.7) | 0.03 |
| Longitudinal Strain (%) | 20.1 (18.9, 21.3) | 22.8 (20.9,24.7) | 21.1 (19.2,23.0) | 0.10 |
| Circumferential Strain (%) | 26.4 (24.5,28.3) | 32.3 (29.2,35.3) | 27.0 (24.3,29.7) | 0.008 |
| ***Diastolic function*** |  |  |  |  |
| E/A | 1.1 (1.0,1.2) | 1.3 (1.1,1.5) | 1.2 (1.0,1.4) | 0.27 |
| e’ (cm/s) | 9.8 (9.1,10.5) | 11.5 (10.4,12.7) | 10.7 (9.6,11.8) | 0.06 |
| E/e’ | 7.6 (6.9,8.3) | 6.7 (5.5,7.8) | 6.4 (5.3,7.5) | 0.15 |
| ***CPET*** |  |  |  |  |
| VO_2max_ (mL/kg/min) | 24.7 (22.8,26.6) | 28.2 (24.9,31.4) | 28.5  (25.5,31.6) | 0.07 |
| Peak RER | 1.2 (1.1,1.2) | 1.1 (1.0,1.3) | 1.1 (1.0,1.3) | 0.95 |
| Resting HR (bpm) | 73.2 (68.5,77.9) | 61.2 (53.7,68.8) | 77.6  (70.1,85.0) | 0.006 |
| Peak HR (bpm) | 148.2 (142.9,153.5) | 143.0  (134.0,152.0) | 153.8 (145.3,162.4) | 0.20 |
| HRR, 1 minute (bpm) | 22.7 (19.4,26.1) | 29.5 (23.9,35.2) | 23.5  (18.1,28.9) | 0.12 |
| CPET=Cardiopulmonary exercise testing; bpm=beats per minute; HR=Heart rate; HRR=Heart rate recovery (defined as the absolute change from peak HR); RER=Respiratory exchange ratio.  For measures of systolic function, diastolic function and CPET, age-adjusted marginal mean (95% CI) estimates based on analysis of covariance are presented. | | | | |

**Supplementary Table 2**: Age-adjusted marginal mean (95% CI) estimates of echocardiography and cardiopulmonary exercise testing measures according to exposure group – Sensitivity analysis excluding participants who received HER2-targeted therapy (N=61)

| **Variable** | ***BRCA1/2* Carriers, Doxorubicin** | ***BRCA1/2* Carriers, No Doxorubicin** | ***BRCA1/2* Non-carriers, Doxorubicin** | **P-value** |
| --- | --- | --- | --- | --- |
| ***Systolic function*** |  |  |  |  |
| Left ventricular ejection fraction (%) | 57.2 (55.1,59.3) | 63.5 (60.3,66.6) | 59.3  (55.9,62.7) | 0.01 |
| Longitudinal Strain (%) | 20.1 (18.9,21.3) | 22.9 (21.0,24.8) | 22.5 (20.2,24.9) | 0.06 |
| Circumferential Strain (%) | 26.4 (24.6,28.4) | 32.3 (29.1,35.3) | 26.6 (23.7,29.5) | 0.008 |
| ***Diastolic function*** |  |  |  |  |
| E/A | 1.1 (1.0,1.2) | 1.3 (1.1,1.5) | 1.2 (1.0,1.4) | 0.37 |
| e’ (cm/s) | 9.7 (9.0,10.5) | 11.5 (10.3,12.7) | 11.0 (9.7,12.3) | 0.04 |
| E/e’ | 7.7 (6.9,8.5) | 6.6 (5.4,7.8) | 6.1 (4.8,7.4) | 0.08 |
| ***CPET*** |  |  |  |  |
| VO_2max_ (mL/kg/min) | 25.2 (23.2,27.3) | 28.0 (24.7,31.3) | 28.5  (24.9,32.1) | 0.21 |
| Peak RER | 1.2 (1.1,1.3) | 1.1 (1.0,1.3) | 1.1 (1.0,1.3) | 0.95 |
| Resting HR (bpm) | 73.2 (68.2,78.1) | 61.8 (54.2,69.4) | 77.8  (69.3,86.3) | 0.012 |
| Peak HR (bpm) | 149.2 (143.7,154.6) | 143.3  (134.5,152.1) | 151.6 (142.0,161.1) | 0.38 |
| HRR, 1 minute (bpm) | 23.6 (20.1,27.2) | 29.3 (23.6,35.0) | 22.6  (16.4,28.8) | 0.18 |
| CPET=Cardiopulmonary exercise testing; bpm=beats per minute; HR=Heart rate; HRR=Heart rate recovery (defined as the absolute change from peak HR); RER=Respiratory exchange ratio.  For measures of systolic function, diastolic function and CPET, age-adjusted marginal mean (95% CI) estimates based on analysis of covariance are presented. | | | | |
